# Toothbrushing and Oral Care Activities of Autistic and Non-Autistic Latino Children

**DOI:** 10.3390/children9050741

**Published:** 2022-05-18

**Authors:** Lucía I. Floríndez, Dominique H. Como, Daniella C. Floríndez, Francesca M. Floríndez, Evelyn Law, Jose C. Polido, Sharon A. Cermak

**Affiliations:** 1 Department of Nursing Research and Quality Improvement, Brawerman Nursing Institute, Cedars-Sinai Medical Center, Los Angeles, CA 90048, USA; 2 The Mrs. T.H. Chan Division of Occupational Science and Occupational Therapy, Herman Ostrow School of Dentistry, University of Southern California, Los Angeles, CA 90089, USA; dcomo@usc.edu (D.H.C.); cermak@chan.usc.edu (S.A.C.); 3 SOS Mentor, Los Angeles, CA 90089, USA; dflorindez@sosmentor.org; 4 Willamette Academy, Willamette University, Salem, OR 97301, USA; fmflorindez@willamette.edu; 5 Department of Occupational Therapy, University of California Irvine Medical Center, Irvine, CA 92868, USA; evelynnnlaw@gmail.com; 6 Division of Dentistry, Children’s Hospital Los Angeles, Los Angeles, CA 90027, USA; jpolido@chla.usc.edu

**Keywords:** oral health, Latinos, toothbrushing, activities of daily living, dental, sensory, autism

## Abstract

Background: Oral care activities, e.g., toothbrushing, are habitual occupations often considered routine. However, for autistic children, performing these routine dental practices can be challenging due to the child’s sensory sensitivities, unique executive function, and the complicated way in which autistic children conceptualize structure and habits. Limited research exists exploring the nuances of oral care routines in the autistic population, and more knowledge is needed to support targeted education interventions to improve oral care and address health inequities. The purpose of this study was to examine videos of oral care routines in the home to understand how oral care activities, such as toothbrushing, were performed by autistic and non-autistic Latino/a children. Methods: Parents/caregivers from eighteen Latino/a families with children between 6 and 12 years old (*n* = 10 autistic children and *n* = 8 non-autistic children) video recorded their child’s oral care routines for three days. The research team blindly coded and analyzed these videos using an oral care observation template to understand how these activities were uniquely performed by the children. Results: Eighty-five oral care videos were analyzed for this study. In addition to noting areas of oral care that can be improved, which included length of brushing and using the correct brushing technique, we identified two themes related to the differences between oral care practices in the autistic and non-autistic children: *parent involvement* and *modifications*. Conclusions: Qualitative findings show that parental involvement was documented especially in the case of autistic children, and that two types of modifications, habitual and sensory, were observed that demonstrated parents being aware of the needs of their autistic child and modifying the oral care activity to meet those needs. By synthesizing observations from the oral care videos into suggestions for practitioners working with families, we hope to supplement knowledge about effective oral care practices for autistic and non-autistic Latino/a children, thereby improving overall oral health and reducing oral health inequities in this population.

## 1. Introduction

Oral health is a vital component of overall health, impacting quality of life both indirectly through the ability to confidently express emotions and facial expressions and directly through fundamental oral functions, such as, chewing and swallowing [1]. Unfortunately, oral health is one of the most prevalent unmet health needs for children in the United States [2,3,4]. Children from underrepresented, medically underserved minority populations or those with special healthcare needs are at even greater risk for experiencing oral health disparities [5,6,7]. No single cause is solely attributable for the disparities in oral healthcare; rather, it is a combination of income, education level, race, disability status, insured status, and other contextual factors [5,8].

Evidence suggests that race/ethnicity and socioeconomic status (SES) are significant factors related to poor oral health. Specifically, Black and Latino people and groups with low SES are at highest risk for caries, oral-related chronic diseases, and poorer overall health status [4,9]. According to CDC reports, the rate of untreated tooth decay for children aged 6–9 years is highest for minorities, 33% of Mexican-American and 28% of Black or African-American compared to 18% of Caucasian children [10]. Recent data have also suggested that Latino/a parents might have limited or incomplete knowledge related to caries risk factors [11,12] and that fatalist beliefs and feelings of stigma or fears related to immigration status and deportation may impact keeping regular dental visits and negatively influence oral health behaviors [11,13,14]. Additionally, it was reported that more than four million children went without needed dental care services because their families could not afford them [15].

In addition to ethnicity and SES, individuals with intellectual and developmental disabilities, such as autistic individuals, are at especially high risk for poor oral health, including having significantly higher prevalence and severity of caries than other populations [16,17,18]. Autistic individuals (we use identity-first language (“autistic child”) in this paper to acknowledge concerns from many in the autism community that person-first language (“child with autism”) perpetuates stigmatization of people on the autism spectrum [19,20]. We also use the terms “Latino/a” in accordance with matching and respecting the ways the participants described themselves) can sometimes experience the world in a way that increases their risk for poorer oral health outcomes [16,21]. This may be the result of difficulties in implementing oral care practices, such as toothbrushing and flossing. Some data suggest that only half of autistic children brush their teeth twice a day, as recommended [22], and up to 73% of parents with autistic children report that toothbrushing, even once a day, is difficult [21]. Practicing in-home oral care routines may be challenging for families due to the behavioral issues exhibited by some autistic children, including repetitive or disruptive behaviors [23]. This challenge may also be exacerbated by sensory sensitivities to the taste or texture of toothpaste or the tactile sensation of the toothbrush bristles in the mouth [21,24]. Moreover, there are minimal clinical protocols designed specifically to meet the needs of autistic patients [25] and a lack of dental professionals willing and capable of working with the population [26].

Although evidence supports the importance of oral health and identifies the challenges faced by autistic children in performing oral care routines, very little research has examined how this issue might be addressed. Furthermore, when intersected with ethnicity, autistic Latino/a children face unique and largely unresearched oral care-related challenges. In current practice, it is unclear what home-based oral care routines look like for Latino/a autistic children, how these routines compare to those of their non-autistic peers, and what oral care methods or modifications are most successful. Additionally, while literature discussing oral health disparities recognizes a need for further research to mitigate oral health inequalities, there have been few studies focused on promoting successful home-based oral care strategies such as toothbrushing and flossing for underserved groups, including autistic children. A recent systematic review of oral care interventions for autistic individuals identified 32 programs aimed at improving dental care experiences. However, only eight of these focused on improving oral care skills and routines in the home, and none were conducted with ethnically diverse participants [27]. Therefore, the purpose of this study was to examine videos of oral care routines in the home to understand the particularities of how oral care activities, such as toothbrushing, were performed by autistic and non-autistic Latino/a children. This information can contribute to a foundation of knowledge upon which to build more effective strategies for completing this important activity of daily living, thereby improving overall oral health and reducing oral health inequities in this population.

## 2. Materials and Methods

This study is a secondary, cross-case qualitative analysis [28] of a larger multi-method study examining the in-home oral care routines of autistic and non-autistic Latino/a children. As part of the larger study, Latino/a families were interviewed to identify the factors that impact their in-home oral care, including how the presence of autism may alter their child’s habits; these data are published elsewhere [7,11,29]. This study focuses on findings from the qualitative portion of the dataset and was approved by the University’s Institutional Review Board (IRB). Prior to engaging in any study activities, all participants provided informed consent.

### 2.1. Participants

Participants in the qualitative study presented in this paper included 18 English- or Spanish-speaking Latino/a families from the Los Angeles, California, area with children between 6 and 12 years old, 10 families with autistic children and eight families with neurotypical non-autistic children. Participants were a snowball sample recruited from the local children’s hospital, from community centers, and via social media. A snowball recruitment strategy was suggested by the IRB to reach out to all possible participants that met eligibility criteria, and the sample size was determined sufficient when saturation of qualitative data was reached. Full recruitment information has been previously published [7,11,29].

### 2.2. Procedures

Parents were asked to video record their child’s oral care activities; these videos were the data source analyzed for this manuscript. Families conducted study-related activities in their preferred language, either English or Spanish. Parents video recorded their child performing their typical oral care routine (e.g., toothbrushing, flossing, using mouthwash) over a three-day period. All parents were provided with the following instructions: “Please film whatever your child does for oral care whenever it happens and include all of their activities.” These instructions were deliberately open ended and left to interpretation by the parent to promote collection of videos that captured pure observation of the occupation. As such, there was not a minimum number of videos necessary to submit. A waterproof, digital camera was offered to participants; however, videos were primarily obtained using a cell phone owned by the parent/caregiver. All videos were saved to a secure memory card using ID numbers to maintain subject confidentiality and to ensure that the coders were blind to child diagnosis and then uploaded to a password-protected electronic database for analysis.

### 2.3. Data Coding

Oral care videos were analyzed using a template coding scheme [30] and cross-case analysis [31]. Prior to starting the coding process, in order to document the unique aspects of oral care between the videos from the autistic and non-autistic children, a template checklist was drafted to facilitate the video coding process and as a means of organizing video content for subsequent interpretation. This template, the Oral Care Observation Guide (OCOG), was created in consultation with pediatric dental professionals and was based on practice standards set forth by the American Academy of Pediatric Dentists for oral care [32]. The OCOG identifies noteworthy details about oral care behaviors including preparation (toothbrush storage, using toothpaste with fluoride, correct amount of toothpaste); brushing (brush at gum line, length of time, attempt to brush all surfaces, etc.); supervision (type of instructions or assistance provided by caregiver); and supplementary oral care habits (use of mouthwash and/or floss). For further details or use of the OCOG, please contact the first author. Videos were coded by two bilingual (English/Spanish) raters blind to the diagnosis of the child using the OCOG. Additionally, the team qualitatively assessed two common components of oral healthcare for children including (1) parent involvement and (2) activity modifications. To help maintain blinding, these raters coded edited videos of only the oral care activity before watching the entire clip and noting the qualitative observations. Any directions or comments made by the parents/caregivers in Spanish were all translated into English for analysis. The research team viewed videos, reviewed any discrepancies in ratings, and discussed trends, resulting in the finalized thematic analysis describing the oral care activities.

### 2.4. Data Analysis

Given our uneven sex and video distribution and overall small sample size, between group (autistic:non-autistic) differences in OCOG ratings, scores from the videos, and differences in proportions were analyzed using only descriptive statistics to present the characteristics of the sample.

## 3. Results

### 3.1. Sample Characteristics

Table 1 provides demographic information about the participants for each group. Of the 10 autistic children, 9 were male and 1 was female. Of the eight non-autistic children, one was male and seven were female. This difference in sex was significant (*p* = 0.0029). Participants in the autistic group had a mean age of 8.8 years (SD +/−2.15). Participants in the non-autistic group had a mean age of 9.00 years (SD +/−2.27).

### 3.2. Videos

In total, 85 videos were received from the 18 participating families—41 from autistic participants and 44 from non-autistic participants. Table 2 details the number of videos the research team received from each family. On average, the families of autistic children submitted 4.1 videos per child (SD +/−1.91), while the families of non-autistic children submitted 5.63 videos (SD +/−3.89). The number of videos across families was not evenly distributed, as some participants submitted as many as 13 videos, and others submitted just one video. Raters had a percent agreement of 94.5%. Disagreements were viewed and scored by members of the research team blind to child diagnosis until consensus was reached.

### 3.3. OCOG Findings

Table 3 describes scores for key features from the Oral Care Observation Guide, comparing videos of autistic and non-autistic children. In the *preparation* section (toothbrush storage, using toothpaste with fluoride, correct amount of toothpaste), autistic and non-autistic children demonstrated similar ability to prepare for brushing their teeth. For example, in the videos that showed the child completing steps prior to brushing, the only area noted to need improvement was the amount of toothpaste being used.

Regarding *brushing* behaviors (brush at gum line, length of time, attempt to brush all surfaces, etc.), some differences between groups were noted for length of brushing and brushing technique. Half of the videos of non-autistic children (50%) and 56.1% of the videos of autistic children indicated children spent 61–120 s or more brushing their teeth. However, five non-autistic children brushed only 0–30 s in 10 videos, while only two videos of autistic children showed such short brushing time. In addition, while all but one autistic child and one non-autistic child attempted to brush at the gumline, more discrepancies were detected in attempting to use circular or brush down motions (vs. raking horizontally across teeth) while brushing. Autistic children (*n* = 8) did not brush with correct technique in nearly half of the videos (*n* = 19), and four non-autistic children in 15% of videos (*n* = 7) did not use correct technique. In the *supplementary oral care habits* section (use of mouthwash and/or floss), the majority (*n* = 14, 78%) of all participants regardless of group failed to implement additional oral care habits. Only two autistic children and two non-autistic children flossed, and only two autistic children and two non-autistic children used mouthwash. Interestingly, only one autistic child *both* flossed and used mouthwash, while no non-autistic children did *both* activities. Lastly, in the *supervision* section (type of instructions or assistance provided by caregiver), autistic children required considerably more caregiver support to complete their dental routines than non-autistic children. For example, 60% of autistic children (*n* = 6) required verbal cues, prompts, or reminders to complete their toothbrushing routines in nearly half of the videos (*n* = 19, 46.3%), which was more than for non-autistic children. Moreover, while caregivers provided instructions to 50% of non-autistic children (*n* = 4), these instructions were only given in 15.9% of videos, and usually appeared only once or twice per child. Lastly, one autistic child needed physical assistance to complete their oral care routine as evidenced in three videos, whereas none of the non-autistic children required this type of support.

### 3.4. Qualitative Observations

In addition to the OCOG, the research team noted two themes in the videos pertaining to oral care practices, including (1) parent involvement and (2) modifications. The different types of parental cues and oral care modifications are presented in Table 4. The first, *parent involvement*, identifies parents as partners in the oral care process, helping to facilitate the oral care activities, either physically or using prompts, verbal cues, or words of affirmation and support. The OCOG identified that 60% of the autistic participants required some sort of involvement from parents, which was was primarily observed as parents providing verbal prompts to clean all surfaces of the teeth or be more thorough in completing the toothbrushing. As one caregiver said to their child (A01), “Don’t forget your top ones (teeth)...Ok? And to rinse again”. Another parent reminded their child (B06) to “...check the braces...”, which prompted the child to pay close attention to the brackets of her braces. Participant A02 received a reminder from his mother to brush his tongue, and to “...remember...go slow, so that you don’t gag...”. At times, verbal cues from the parent were a little more subtle, “Are you done...is there anything else you do?” (A01). This gentle nudge prompted the child to complete the next two steps of his oral routine, which were using floss and mouthwash. Beyond ensuring that children attempted to clean all surfaces of their mouth, sometimes the children required additional prompts to initiate oral care activities. As evidenced in one observation, the child (A03), was in the other room when the mom called out to the child that it was, “...time to brush your teeth”; the child required a couple of reminders to get started. While not common within this sample, there was one participant (A09) who required hand-over-hand physical assistance from his caregiver to ensure that his teeth were thoroughly brushed. During his attempt to complete his oral care routine on his own, he brushed his teeth independently for approximately 20 s, at which point his mother took the toothbrush in hand and brushed the tooth surfaces that the child missed.

The other theme observed by the research team was *modifications*, which describes the habitual and sensory modifications parents/caregivers enacted to assist their child with the oral care activity, uniquely observed in the autistic participants. Habitual modifications include using tools such as timers or electric toothbrushes with timers to ensure brushing happened for the required amount of time and were used to help establish successful oral care routines for the children. Forty percent of autistic children regularly used timers/timekeeping in their toothbrushing routines. For example, one autistic child (A01) could not start his routine without the timer present, as evidenced in videos where he would leave the room to grab the timer and bring it to the bathroom, and used it to monitor how long to brush his teeth. Another participant (A03), kept time while brushing his teeth by humming a song. When he did this, he was able to maintain a longer duration for brushing his teeth, consistently brushing for more than 60 s. Participant A06 was also observed to hum and keep time while brushing his teeth, and participant A04 used a *different* toothbrush belonging to a sibling to play a song as a timer while he used his own non-song-playing toothbrush on his teeth.

Sensory modifications were used to alter the context and environment to make the toothbrushing setting more tolerable. For example, participant A03 was only willing to brush his teeth while standing on the counter, often positioning himself in such a way that he could not see himself in the mirror, possibly so he could eliminate as much visual stimuli as possible. He remained crouched in this position on the counter, avoiding the mirror until the task was complete. This participant was shown standing and sitting on the counter, avoiding the mirror, in all his videos. While participant A03 appeared to be trying to limit his visual sensory input, he also was observed to be standing on his tiptoes until his toothbrushing was complete, perhaps providing himself with additional proprioceptive input. Participant A01 was noted to have a lot of excessive movements while brushing his teeth, including swaying, pacing, and shuffling his feet side to side. It also was noted that the location for completing oral care routines varied for some of the participants, as did the level of lighting. While all videos were recorded in the bathroom, a couple of the participants deviated from standing in front of the sink and preferred to brush their teeth in front of a full-length mirror (A06) or in the bathtub (A09). Other participants were noted to stand in bathrooms with dimmed or no lighting (A01, A03, A06, A10), reducing the stimulus from overly bright lighting.

## 4. Discussion

An important finding of our work indicates that for Latino/a autistic children, parents play a significant role in their child’s ability to access their in-home oral care. However, this study also highlights that some aspects of the routine were completed easily, independently by most participants. For example, nearly all the participants were able to complete the steps necessary to prepare for toothbrushing, such as placing the correct amount of toothpaste on the brush (*n* = 8 autistic and *n* = 8 non-autistic). Additionally, half of the participants also brushed for the two minutes suggested by the AAPD. In fact, the autistic children more frequently brushed for at least two minutes as compared to their typically developing counterparts. This may be the result of modifications utilized and/or the strategies implemented by parents involving timers to help regulate the activity. Families and practitioners can take from this finding that independence and autonomy in toothbrushing is a possibility for some autistic children once certain needs are met and accommodations made to help understand the autistic neurotype.

This study also highlights several areas for improvement in toothbrushing. Firstly, as indicated above, brushing for the recommended time and using the correct technique can be improved, including using timers and providing periodic supervision or modeling of the activity. This was noteworthy in the non-autistic children who were sometimes observed brushing their teeth for 30 s or less (*n* = 10 children). These children were not supervised by their parents/caregivers and completed their oral care routines with no feedback or input on their activity. Next, increased attention on incorporating flossing and mouthwash into oral care routines will enhance these habits. Although flossing and using mouthwash before or after brushing are included in the AAPD guidelines in children with increased risk of caries (AAPD, 2016), parents often see them as optional, supplemental tasks. This was observed for both parents of autistic and non-autistic children, as only three children either flossed and/or used mouthwash and did so sporadically. However, in these populations at increased risk of caries or with previous caries activity, they are important components of the dental routine. A recent survey indicated that Latino/a parents/caregivers with lower socioeconomic status, fewer years of education, and previous poor experiences at the dentist had lower overall oral care knowledge than their peers with higher incomes and more years of education [11]. Therefore, it is possible that Latino/a parents/caregivers would benefit from more education about the significance of the role of flossing and mouthwash in oral health. Parents might also benefit from strategies for how to incorporate these additional tasks into the existing routine. Additionally, upwards of 70% of parents of autistic children report that toothbrushing is a challenge, and it is possible that some Latino/a parents with autistic children might feel increased stress with adding more to this already taxing activity. Occupational therapists and healthcare workers, in collaboration with dental providers and caregivers, are well suited to help parents and children develop a plan of action to incorporate these additional components of flossing and using mouthwash into their existing activities of daily living.

Moreover, the role of parents and their involvement in their children’s oral care routines was something highlighted in this study. Latino/a parents and caregivers have previously been reported to prioritize the oral care needs of their children over their own [7,11], and parental involvement was observed especially in the case of autistic children. Two types of modifications, habitual and sensory, were observed that demonstrated parents being aware of the needs of the child and modifying the activity to meet those needs. Most of the non-autistic children did not need to make any modifications to their toothbrushing and oral care routines. Only one participant (B01) used a timer, on occasion, to monitor her time, while another participant (B04) watched a video online during toothbrushing. Conversely, all the autistic children utilized some type of modification, whether it was sensory or habitual. Parents of autistic children also utilized verbal cues and prompts in most of the videos submitted.

Practitioners can aid parents in improving oral care routines by providing parent/caregiver education about when to use prompts and what type of cues would be most beneficial, and how to gradually reduce their use to encourage self-reliance, when appropriate. Dental practitioners in collaboration with healthcare workers such as occupational therapists can aid parents in identifying their child’s sensory sensitivities (i.e., taste of toothpaste, feel of the bristles, vibration of the electric toothbrush, etc.) and create an individualized plan to adapt the activity for the child’s needs. This extends to the exploration of habitual modifications that might suit the needs of the families, such as the use of timers or videos on an electronic device to keep time (and the child entertained) as was observed. Indeed, previous work has indicated that video modeling of oral care activities for autistic children has had positive outcomes on improving oral hygiene and skills at home [33,34,35]; these interventions should be expanded and continued.

Situating toothbrushing as a dynamic relationship of occupational form, developmental structure, meaning, and purpose [36], we hope to shed light on the occupational performance of oral care in autistic and non-autistic Latino/a children. The occupational form, or the objective set of physical and sociocultural circumstances, is different for each group and for each child. Moreover, everyday occupations, such as toothbrushing, are “seen but unnoticed” aspects of our existence [37]. Therefore, it is important to aid individuals in their pursuit of competency in everyday occupations, including oral care activities. Although these activities such as toothbrushing are often perceived as predictable, performing these routine oral care activities can be challenging [21], and autistic individuals often need additional assistance to complete certain tasks related to oral care [38]. Autistic children, especially those of Latino/a descent, face several oral health disparities that make access to and engagement in oral care occupations challenging [7]. Our study corroborates these previous findings, and indicated that Latino/a autistic children have sensory issues related to oral care activities. Addressing these issues by incorporating modifications or adaptations such as we observed in some participants may facilitate oral care [16]. This study also indicates that accessing and performing oral care routines often requires Latino/a autistic children and their families to work together and that non-autistic Latino/a children need to receive periodic supervision to ensure they perform the routine as instructed. Occupational therapists and healthcare workers can aid families in their pursuit of better oral health through improving their dental care routines, acting as liaison between family and dentists, and by serving as members of multidisciplinary teams that help extend the understanding of working with this medically underserved population.

### Limitations

This analysis has several limitations. Firstly, the authors want to be careful to acknowledge the small sample size and that these findings may not be reflective of all groups of Latinos/as, nor should they be assumed to be standards by which to understand Latino/a or autistic populations. As this was a snowball sample, the representativeness of the sample is not guaranteed, as is evidenced by the uneven distribution of the sex of participants across each group. However, as this population (autistic and non-autistic Latino/a children) is typically considered underrepresented in research or hard to access, we heeded the guidance of our IRB to utilize a snowball recruitment method and wanted to be sensitive to including all parties in our sample who were interested and eligible to participate until we reached saturation of qualitative findings, despite resulting in uneven sex distribution across groups. In addition, while performing comparative statistics on our sample is of limited usefulness, we firmly believe that lack of statistical significance does not rule out the existence of meaningful findings when a sample size is small. In our case, the modifications of the oral care activities observed between groups can be used as important data to inform clinicians. However, future work should consider matching the distribution characteristics of the final sample to improve statistical quality.

Second, we did not collect data from non-Latino/a participants, so we have no comparisons to make to other populations outside of our sample. Third, since families were encouraged to film their child’s real-life oral care routines without much direction, videos were intended to be used as observations only, and not as clinical evaluation tools. Additionally, though we took steps to reduce the likelihood that an autism diagnosis could be ascertained from the video clips during the blind oral care activity coding, it is possible that raters could determine the diagnosis of the participants due to behavioral or verbal cues. Maintaining full blinding to groups is difficult, and future work should consider how to improve this step of the coding process.

Next, the autism diagnosis was based on parent-report of a previous clinical diagnosis and not confirmed by the research team. Therefore, we make no conclusions about how aspects of the autism diagnosis (behavior, etc.) may have impacted ability or participation in the observed oral care activities. Moreover, sex distributions in our sample were uneven and may mask autistic and non-autistic differences. For example, a study team in Lithuania found that girls more often than boys regularly brushed their teeth and completed supplemental oral care activities such as flossing [39]. Further research on gender- and sex-based differences in autism and oral care is needed. Finally, although the study team consulted with dentists in developing the OCOG, the study coders were not established dentists/oral care experts.

## 5. Conclusions

As a daily occupation tied to overall health and wellbeing, oral care activities deserve and require more attention. Primarily, understanding in-home oral care routines may contribute to addressing oral care disparities in autistic and non-autistic Latino/a children. The process of exploring the particularities of routines involves addressing the occupation of oral care itself, while also considering the influence of the cultural contexts, family and child descriptors, performance patterns, and systemic restrictions on the activity. By synthesizing components of oral care routines into suggestions for successful practice, we hope to begin the discussion on effective oral care strategies for autistic and non-autistic Latino/a children. Additional research is needed to see if the oral care routines observed in this study are applicable to other segments of the Latino/a population.

## Figures and Tables

**Table 1:** Demographic information of sample.

Characteristics	Autistic Children (*n* = 10)	Non-Autistic Children (*n* = 8)
Language spoken by caregiver		
English	70% (7)	50% (4)
Spanish	30% (3)	50% (4)
Primary caregiver’s gender		
Female	90% (9)	100% (8)
Male	10% (1)	0
Years of education of participating parent	13 (6–19)	13.9 (7–17)
Age of child	9.0 (6–12)	7.9 (6–12)
Gender of child		
Female Male	10% (1) 90% (9)	87% (7) 13% (1)

Data are presented as mean (range) for age and years of education and percentage (*n*) for language, caregiver’s gender, and child’s gender.

**Table 2:** Participant age, gender, and video information.

Autistic Group (*n* = 10)			Non-Autistic Group (*n* = 8)		
Participant ID	Age and Gender	No. of Oral Care Videos	Participant ID	Age and Gender	No. of Oral Care Videos
A01	7, M	5	B01	7, F	13
A02	12, M	4	B02	11, F	9
A03	8, M	7	B03	11, F	1
A04	8, M	1	B04	7, F	6
A05	11, M	5	B05	12, M	3
A06	8, M	5	B06	8, F	6
A07	7, F	5	B07	6, F	4
A08	9, M	1	B08	10, F	2
A09	6, M	3			
A10	12, M	5			
	9 (mean)	41 videos		7.9 (mean)	44 videos

**Table 3:** Frequency of key features from the Oral Care Observation Guide by autistic and non-autistic children.

	Autistic Children		Non-Autistic Children	
	Individuals (*n* = 10)	Videos (*n* = 41)	Individuals (*n*= 8)	Videos (*n* = 44)
Preparation				
Yes–Was too much toothpaste used?	2 (20%)	4 (9.7%)	1 (12.5%)	1 (2.3%)
Brushing				
Length of time child brushed teeth (seconds)				
0–30	2 (20%)	2 (4.9%)	5 (62.5%)	10 (22.7%)
31–60	6 (60%)	16 (39%)	6 (75%)	12 (27.3%)
61–120	5 (50%)	10 (24.4%)	4 (50%)	17 (38.6%)
More than 120	4 (40%)	13 (31.7%)	2 (25%)	5 (11.4%)
No–Did the child attempt to brush all surfaces?	2 (20%)	4 (9.7%)	1 (12.5%)	1 (2.3%)
No–Did the child use circular brushing motions?	8 (80%)	19 (46.3%)	4 (50%)	7 (15.9%)
Supplementary Oral Care Habits				
Yes-Child used floss	2 (20%)	5 (12.2%)	2 (25%)	13 (29.5%)
Yes-Child used mouthwash	2 (20%)	6 (14.6%)	2 (25%)	9 (20.5%)
Supervision				
Caregiver provided verbal instructions/prompts/cues on toothbrushing	6 (60%)	19 (46.3%)	4 (50%)	7 (15.9%)
Caregiver provided physical assistance to child to complete toothbrushing	1 (10%)	3 (7.3%)	0 (0%)	0 (0%)

Data are presented as *n* (percentage).

**Table 4:** Summary of observed oral care adaptations.

Group	Parent Instructions/Assistance	Parent Support Quotes	Adaptations/Behaviors/Equipment Used
Autistic children (*n* = 10)	Verbal reminders/instructions: 24/41 videos Physical Assistance: 2/37 videos	“Good job with getting that side extra, that was the side that you had the cavity.” “Don’t drink it okay? We just swish it” “Don’t spill.” “Don’t forget your top part, ok?” “Are you done? Is there anything else that you’re supposed to do?” “What else do you have to do? “Remember to stick your tongue out, that way you’re not gagging, ok?” “That’s good baby. You’re doing better with that.” “Come on, do a good job brushing.” “Did you rinse off your toothbrush?” “Did you put the lid back on the toothpaste?” “Ok, wash your mouth.” “Show your toothbrush. Come on show it.” “Make circular motion with your toothbrush.” “Rinse your mouth.” “Show me how much [toothpaste] you’re putting on.” “Why don’t you like flossing? Why don’t you do that [floss]?” “Remember, little circles.” “Your other molar is growing in the back.” “Careful!” “Brush your tongue.” “Wash with water.”	1/10 children: strict adherence to hourglass sand timer 1/10 children stood on the sink counter to brush teeth 2/10 children used electric toothbrush 3/10 children hummed throughout toothbrushing 1/10 children required rest breaks 1/10 children played music while brushing teeth
Non- autistic children (*n* = 8)	Verbal reminders/instructions: 11/44 videos Physical Assistance: 0/47 videos	“Make sure you get the bottoms too.” “What about dental floss?” “Don’t forget the mouthwash.” “Not too hard.” (about child’s toothbrushing) “Did you check in the front?” (on flossing) “Up and down.” (on teeth) “Check your braces.” “No, you don’t do another toothpaste.”	2/8 children used electric toothbrush 1/8 children brushed teeth 2x consecutively

## Data Availability

For more information on the sample or for inquiries about use of the OCOG, please contact the first author.
